# Population Genetic Diversity of Two Blue Oat Mite Species on *Triticum* Hosts in China

**DOI:** 10.3390/insects14040377

**Published:** 2023-04-12

**Authors:** Xian Wang, Wenjie Wang, Yang Qin, Mian Wang, Yaying Li, Huai Liu

**Affiliations:** Key Laboratory of Agricultural Biosafety and Green Production of Upper Yangtze River, Key Laboratory of Entomology and Pest Control Engineering, College of Plant Protection, Southwest University, Chongqing 400716, China

**Keywords:** *Penthaleus major*, *Penthaleus tectus*, molecular diversity, mitochondrial COI, population genetic structure

## Abstract

**Simple Summary:**

Blue oat mite species mainly include *Penthaleus major*, *P. falcatus*, *P. tectus*, and *P. minor*. Among them, *P. major*, *P. falcatus* and *P. tectus* are important pest mites on gramineous crops, which often occur in low-temperature and high-humidity environments. These three mites are difficult to distinguish in the field due to their small size and similar shape. Furthermore, they exhibit different resistance to chemical pesticides, making it challenging to control their outbreak. Worryingly, we only saw reports of frequent occurrence of *P. major* in winter wheat regions in China, and no other species of blue oat mites were found. Understanding the distribution pattern, genetic diversity, and genetic differentiation among populations of blue oat mite species in winter wheat regions of China is essential for developing effective control programs. In this study, we evaluated the distribution of blue oat mites in the major wheat regions in China and assessed the level of genetic diversity and genetic structure of blue oat mites from winter wheat regions of China using mitochondrial cytochrome c oxidase I (COI) sequences.

**Abstract:**

Blue oat mite species, including *Penthaleus major* and *P. tectus*, are pests widely distributed across China that cause damage to winter wheat. This study evaluated the genetic diversity of *P. major* and *P. tectus* on *Triticum* hosts collected from 23 geographic locations based on mitochondrial cytochrome c oxidase subunit I (COI) sequences. We identified nine haplotypes in 438 *P. major* individuals from 21 geographic locations and five haplotypes in 139 *P. tectus* individuals from 11 geographic locations. Meanwhile, *P. major* exhibits high values of haplotype diversity (*Hd*) and nucleotide diversity (*Pi*) (*Hd* = 0.534 > 0.5 and *Pi* = 0.012 > 0.005), representing a large stable population with a long evolutionary history. *P. tectus* shows low values of *Hd* and *Pi* (*Hd* = 0.112 < 0.5 and *Pi* = 0 < 0.005), which suggest recent founder events. Moreover, demographic analysis suggested that *P. major* and *P. tectus* have not undergone a recent population expansion. The lowest genetic variation was observed in Xiangzhou (XZ-HB), Zaoyang (ZY-HB), Siyang (SY-JS), and Rongxian (RX-SC), with only one species and one haplotype identified in over 30 individuals. Robust genetic differentiation was found in *P. major* compared to *P. tectus*, which provides a theoretical basis for the widespread distribution of *P. major* in China.

## 1. Introduction

Blue oat mites (*Penthaleus* spp.) are significant agricultural pests in temperate regions around the world, attacking various pastures, vegetables, and crop plants [1,2,3,4,5,6]. These mites cause severe damage to plants by directly penetrating the plants’ epidermal cells and removing the cellular contents [7,8]. Among the three blue oat mites, *P. major* was first described by Dugés in France. In addition, Womersley gives this mite its current name, *P. major*, by recognizing the synonymy among previously named specimens [9]. After that, two other asexual mites were found, including *P. falcatus* and *P. tectus* [8,10,11,12].

Previous research has shown that *P. major*, *P. falcatus*, and *P. tectus* were diploid and reproduced by thelytokous parthenogenesis [10,11]. A high-level clonal variation was found within populations of *P. major* from southeastern Australia [11,13,14], while *P. falcatus* and *P. tectus* have fewer allozyme clones, six and four, respectively [11]. *P. major* has been studied as a model species to investigate how genetic diversity is maintained in asexual organisms [15].

The extent of genetic variability of a population determines its ability to adapt to the environment [16,17]. Numerous correlations between genetic variation and fitness traits, such as growth, survival, and resistance have been reported in the literature [18,19,20]. Recently, the genetic diversity of different geographical populations of pests has been widely studied based on mitochondrial cytochrome c oxidase subunit I (COI) sequences, including *Aedes albopictus* [21] and *Culicoides mahasarakhamensee* [22].

In China, two pest mites are widely reported on wheat, including *P. major* Dugés (Acari: Penthaleidae) and *Petrobia latens* Müller (Acari: Tetranychidae). *P. major* is mainly distributed in 29–37° N, including southern Hebei, Shanxi, Shandong, Henan, Anhui, Jiangsu, Zhejiang, and Sichuan. *P. latens* is mainly distributed in 34–43° N, including Liaoning, Beijing, Gansu, Qinghai, Xinjiang, Xizang, Hebei, Henan, Shandong, Shanxi, Shaanxi, and Anhui [23]. Most domestic research has primarily focused on the harm of mites on winter wheat and a series of control methods [24]. Foreign studies show that there are at least three different species in the same genus for blue oat mites, and there is a phenomenon of simultaneous damage [4,25]. Furthermore, different types of pest mites have significantly different resistance to chemical agents [11,26].

Winter wheat is widely planted in China and is always damaged by pest mites. However, the specific species of mites on wheat are still unclear in China. Thus, rigorous research is needed to learn about the distribution pattern and genetic diversity of mites in different parts of the country. Therefore, in this study, we aimed to investigate the damage of blue oat mites in three major winter wheat regions, including Southwestern Winter Wheat Zone (SW), Yellow and Huai River Facultative Winter Wheat Zone (YH), and Middle and Low Yangtze Valley Winter Wheat Zone (YV). Furthermore, we analyzed the genetic diversity of blue oat mites by mitochondrial COI sequences. This study aimed to examine the genetic variation and structure of blue oat mites on Triticum hosts from different locations across China using mitochondrial DNA. Additionally, phylogenetic relationships of haplotypes with published haplotypes of COI worldwide were investigated.

## 2. Materials and Methods

### 2.1. Investigation on Wheat Mites

This study conducted a field survey of important pest mites on winter wheat in major regions across China in December 2018 and February–March 2019. The survey covered 10 provinces and one municipality, namely, Sichuan, Guizhou, Yunnan, Jiangsu, Anhui, Hubei, Henan, Shanxi, Shandong, Shaanxi, Hebei, and Chongqing. Specifically, 35 survey sites were included, namely Rongxian, Mianzhu, Santai, Nanchong, Tongnan, Liangping, Rongchang, Qianxinan, Liupanshui, Baoshan, Lijiang, Suining, Donghai, Xuzhou, Jiawang, Siyang, Funnan, Bozhou, Changfeng, Xiaoxian, Dangyang, Xiangzhou, Zhongxiang, Zaoyang, Dancheng, Shanzhou, Tanghe, Luohe, Linyi, Ruicheng, Gaotang, Weinan, Xingtai, Handan, and Yuncheng.

### 2.2. Specimen Collection

A total of 577 adult mites were collected from Triticum hosts at 23 locations across China between December 2018 and February–March 2019. According to previous academic reports, the 23 locations were divided into three natural geographic groups, respectively, Southwestern China (SW), Middle and Lower Yangtze Valleys (YV), and Yellow and Huai Valleys (YH). The mites were stored in 95% ethanol or at −20 °C until DNA extraction. The sampling locations were distributed in seven provinces, including Sichuan province, Jiangsu province, Anhui province, Henan province, Shandong province, Hubei province, Shanxi province, and one municipality, Chongqing.

### 2.3. DNA Extraction and Mitochondrial COI Gene Amplification

DNA was extracted from individual female adult mites using 30 μL STE buffer in a 1.5 mL centrifuge tube. To the extract, 2 μL proteinase K (20 mg/mL) was added, and the mixture was incubated at 37 °C for 30 min, followed by heating to 95 °C for 5 min [27,28]. COI fragments were amplified using COI universal primer pair, LCO1490: 5′-GGTCAACAAATCATAAAGATATTGG-3′ and HCO2198: 5′-TAAACTTCAGGGTGACCAAAAAATCA-3′ [29]. Polymerase chain reaction (PCR) mixture (25 μL) contained 2.0 μL supernatant of the lysate, 1.0 μL of each primer (10 μM), 2.0 μL of dNTPs (2.5 mM), 2.0 μL of Mg^2+^ solution (25 mM), 2.5 μL of 10 × PCR buffer (Mg^2+^ free), 14.5 μL nuclease-free water, and 0.25 μL of rTaq polymerase (TaKaRa). The PCR reactions procedure was as follows: initial denaturing for 5 min at 95 °C; 35 cycles of 95 °C for 60 s, 57 °C for 60 s, and 72 °C for 90 s; and a final extension at 72 °C for 5 min. The size and the quality of PCR products were examined by 1.0% TAE agarose gel electrophoresis and sequenced in both directions by Sangon (Shanghai, China).

### 2.4. Data Analysis

The assembling and alignment of COI sequences were completed in DNASTAR LASERGENE 7.1.0 and MEGA 7.0, respectively [30,31,32]. Genetic diversity parameters were determined in DnaSP 5.10.01 [27], including the number of polymorphic sites (*S*), the total number of mutations (η), the number of haplotypes (*H*), haplotype diversity (*Hd*), nucleotide diversity (*Pi*), and the average number of nucleotide differences (*K*). A total of 12 haplotypes reported from Canada and Poland were used to analyze the phylogenetic relationships. Phylogenetic analyses were performed in MEGA 7.0 using the Neighbor-Joining method, while haplotype networks of *P. major* and *P. tectus* haplotypes were constructed in TCS 1.21 [33]. Pairwise *F_ST_* and gene flow (*N_m_*) between each pair of the sampled locations and pairwise *F_ST_* and gene flow (*N_m_*) among three geographic regions were calculated in ARLEQUIN 3.5 [34]. The mismatch distribution analysis was performed to detect historical population expansion events in *P. major* and *P. tectus* populations in DnaSP 5.10.01. A multimodal pattern implies that populations are at demographic equilibrium, whereas a unimodal pattern shows that populations are experiencing rapid demographic growth [35,36].

## 3. Results

### 3.1. Distribution Pattern of Wheat Pest Mites

**Figure 1:**
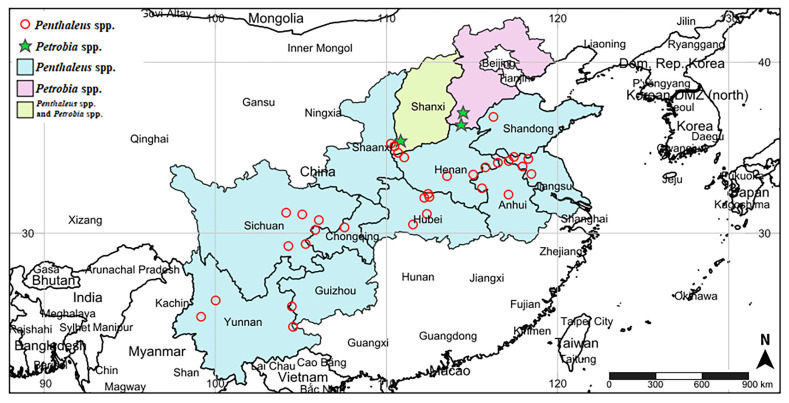
Investigation map of pest mites on wheat in main winter wheat areas of China, including survey areas and specific survey sites. The picture was created using the online website SimpleMappr (https://www.simplemappr.net accessed on 16 December 2022) with latitude and longitude data.

Two genera of mites were found in the main winter wheat regions of China during this survey, including *Penthaleus* spp. and *Petrobia* spp. (Figure 1). The study found that *Penthaleus* spp. were the main pest mites in winter wheat regions and were distributed in 11 provinces and a municipality, including Yunnan, Sichuan, Guizhou, Shaanxi, Hubei, Shanxi, Henan, Anhui, Shandong, Jiangsu, and Chongqing. The range of *Petrobia* spp. was small, primarily in Shanxi and Hebei. Moreover, the map revealed that both *Penthaleus* spp. and *Petrobia* spp. occurred in Shanxi Province. *Penthaleus* spp. was the most widely distributed; however, it was not found in Hebei. Among the 35 survey sites, *Penthaleus* spp. was found in 32 sites, accounting for up to 91.43%, while *Petrobia* spp. was only found in three sites. The population distribution range and number were significantly lower than *Penthaleus* spp.

### 3.2. Population Distribution of Blue Oat Mites

**Figure 2:**
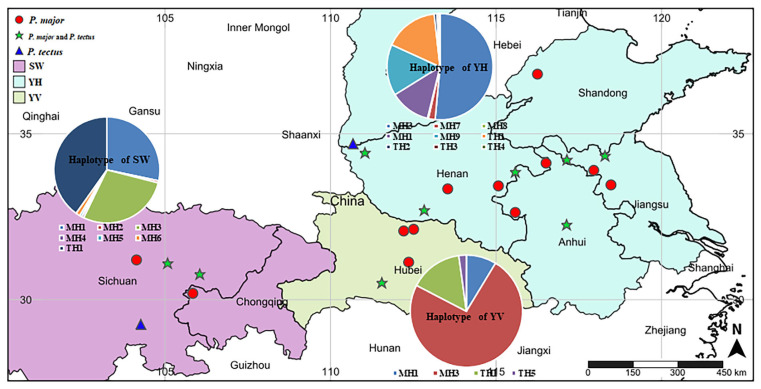
Location map of the studied wheat populations of *P. major* and *P. tectus* in Southwestern China (SW), Middle and Lower Yangtze Valleys (YV), and Yellow and Huai Valleys (YH), which are the three major winter wheat areas of China. The picture was created using the online website SimpleMappr (https://www.simplemappr.net accessed on 16 December 2022) with latitude and longitude data. The haplotype network diagram was created in PowerPoint.

In this study, the dominant population of blue oat mite species on winter wheat in China was identified (Figure 2; Table 1). Two species of blue oat mites were found in 577 samples, including *P. major* and *P. tectus*. Among them, *P. major* accounted for 75.91%, more than *P. tectus*. *P. major* and *P. tectus* were found throughout three natural geographic groups, including Southwestern China (SW), the Middle and Lower Yangtze Valleys (YV), and the Yellow and Huai Valleys (YH). Of the 23 sites surveyed, *P. major* was found in 21 survey sites, with the exceptions being Rongxian (RX-SC) and Ruicheng (RC-SX). The distribution of *P. tectus* is similar to that of *P. major*, but it is not always found in the same sites. At nine sites, *P. major* and *P. tectus* co-occurred, including Santai (ST-SC), Nanchong (NC-SC), Dangyang (DY-HB), Xuzhou (XZ-JS), Donghai (DH-JS), Bozhou (BZ-AH), Changfeng (CF-AH), Shanzhou (SZ-HN), and Tanghe (TH-HN). Additionally, only *P. tectus* was found at two survey sites, including Rongxian (RX-SC) and Ruicheng (RC-SX).

### 3.3. Haplotype Diversity, Nucleotide Diversity, and Haplotype distribution

The COI gene fragment, which was amplified from 577 individuals in 23 different locations across three central winter wheat regions, ranged from 657 to 658 bp (Table 2). Of the 438 individuals of *P. major*, nine haplotypes (MH1-MH9) were identified with 22 polymorphic nucleotide sites. Nineteen of these sites were parsimony-informative, and three were singleton sites. The A/T content was significantly higher (70.30%) than the C/G content (29.70%). Meanwhile, 139 *P. tectus* individuals generated five haplotypes (TH1-TH5) with four polymorphic nucleotide sites. Four of these sites were parsimony-informative sites and none were singleton sites. The A/T content was significantly higher (69.90%) than the C/G content (30.10%).

The *Hd* of *P. major* ranged from 0.000 in the seven populations (NC-SC, XZ-JS, SY-JS, BZ-AH, XZ-HB, ZY-HB, TH-HN) to 0.708 CF-AH population (Table 3). Haplotype diversity of *P. major* was identified in 12 of the 21 locations. For the other nine locations, only one haplotype was found. The *Hd* of *P. tectus* ranged from 0.000 in the six populations (RX-SC, ST-SC, NC-SC, XZ-JS, SZ-HN, TH-HN) to 0.467 CF-AH population. Additionally, haplotype diversity of *P. tectus* was only observed in three of the 11 locations. For the other eight locations, only one haplotype was found. Furthermore, for all individuals, *P. major* had large *Hd* and *Pi* values (*Hd* > 0.5 and *Pi* > 0.005), indicating a large stable population with an extended evolutionary history. In contrast, *P. tectus* had smaller *Hd* and *Pi* values (*Hd* < 0.5 and *Pi* < 0.005), implying recent founder events [37,38,39,40].

### 3.4. Phylogenetic Relationship and Haplotype Network

**Figure 3:**
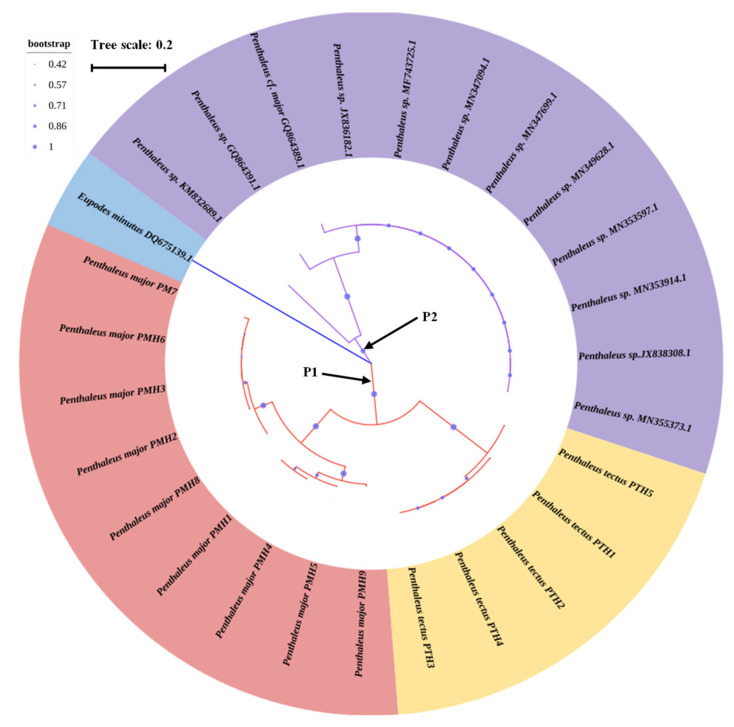
The Neighbor-Joining phylogenetic relationship of 9 haplotypes of *P. major*, 5 haplotypes of *P. tectus*, and 12 haplotypes of *P*. sp. The scale bar indicates the average number of nucleotide substitutions per site. The picture was first drawn in MEGA 7.0, and then the picture was adjusted through the online website (https://itol.embl.de/ accessed on 24 March 2023).

The Neighbor-Joining method was used to construct the phylogenetic tree with COI sequences of *P. major*, *P. tectus,* and 12 published COI sequences of *Penthaleus* sp. (Figure 3). Among the 26 haplotypes of *Penthaleus* sp., Clade P1 (PMH1-PMH9 and PTH1-PTH5 from China) and Clade P2 (other samples from Canada and Poland) were identified. The COI sequence of *Eupodes minutus* with GenBank accession number DQ675139.1 was chosen as the outgroup. The phylogenetic tree showed that the *Penthaleus* sp. in China were divided into two branches distinct from those in Canada and Poland, while the 14 haplotypes in China were split into two branches, representing *P. major* (PMH1-PMH9) and *P. tectus* (PTH1-PTH5), respectively.

**Figure 4:**
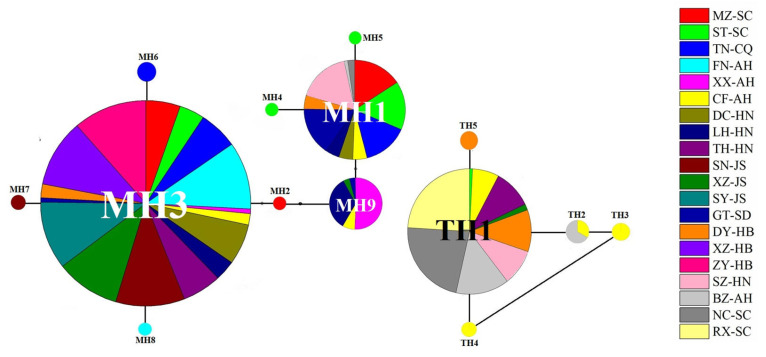
The TCS network for haplotypes of *P. major* and *P. tectus* populations based on the sequence of the COI gene. The haplotype network diagram was constructed in TCS 1.21, and then the color was added in PowerPoint.

The haplotypes of *P. major* and *P. tectus* populations are shown by the TCS network, with varying colors and sizes of circles representing different sampling sites and the number of individuals detected. MH3 is the most crucial haplotype for *P. major*, and TH1 is the most abundant haplotype for *P. tectus* (Figure 4).

### 3.5. Population Differentiation and Genetic Structure

Population pairwise fixation index (*F_ST_*) and gene flow (*N_m_*) investigated the diversity in 21 *P. major* populations and 11 *P. tectus* populations (Table 4 and Table 5). The pairwise *F_ST_* tests of *P. major* populations indicated significant differentiation in 120 of 210 location pairs based on mitochondrial COI gene.

Gene flow between SW and YV of *P. major* and between SW and YH of *P. tectus* show a relatively low degree due to the low *N_m_* value (*N_m_* < 1), suggesting that genetic drift might result in genetic differentiation (Table 6 and Table 7). For three *P. major* natural geographic groups, higher gene flow (*N_m_* > 1) was found between SW and YH, while for *P. major* and *P. tectus*, high gene flow (*N_m_* > 3) was found between YV and YH. In addition, for *P. tectus*, high gene flow (*N_m_* = 4.23) was also found between SW and YV.

AMOVA results of *P. major* indicated significant molecular genetic variations among the locations within geographic regions (55.74%) (Table 8). The variation within sites was 42.20%. Only 2.05% variation was found among geographic regions. However, for *P. tectus*, the AMOVA results indicated that a significant portion of the molecular genetic variation was found within locations (88.43%). The variation among the sites within geographic regions was 9.21%. Only 2.35% variation was found among geographic areas.

### 3.6. Demographic Analysis

**Figure 5:**
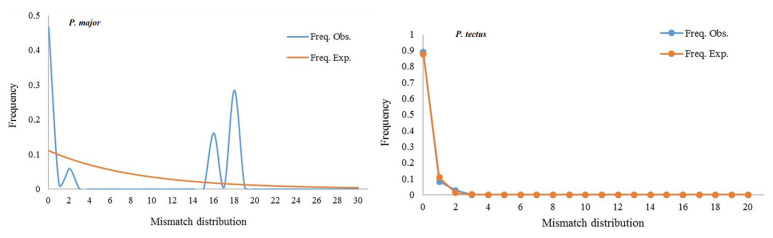
Mismatch distribution analysis of *P. major* and *P. tectus* from 23 locations based on COI gene.

Demographic analysis showed that the overall mismatch distribution for all sampled localities showed the multimodal profile (Figure 5), suggesting that *P. major* populations in China might not have undergone a sudden demographic expansion. The actual observation results are compatible with the predicted constant model when assuming a constant population size for *P. tectus*, demonstrating that the population status is consistent with the expected model assumption. Altogether, results suggested that the population size of *P. major* and *P. tectus* have not changed over time.

## 4. Discussion

Blue oat mites, which are widely distributed in southern Australia, are an important agricultural pest species in Australia [4,5]. Electrophoresis results suggest that there are differences between species in terms of clonal diversity [11,13,14]. Previous research has also showed that *Penthaleus* spp. respond differently to pesticides [26,38]. Therefore, clarifying the distribution range and population density of different species is essential for adequate control.

In this study, *Penthaleus* spp. and *Petrobia* spp. were found on winter wheat, consistent with previous research reports [23]. A large number of blue oat mites (*Penthaleus* spp.) were found in three major winter wheat regions of China, including *P. major* and *P. tectus*. The distribution range and population density of *P. major* were broader and higher than *P. tectus*, suggesting that controlling pest mites on wheat is very difficult because different species have different resistance levels. The risk is that the sympatric occurrence of different species will make control more difficult. We also found high levels of genetic diversity in the *P. major* population by analyzing mitochondrial cytochrome oxidase subunit I sequences. The distribution proportion of *P. major* was significantly higher than *P. tectus*, and the total number of *P. major* was as high as 75.91% in 577 samples, consistent with the study of Australian scholars [41]. *P. tectus* only occurs in limited areas and is recorded only from Australia and South Africa [12]. In China, *P. tectus* was first recorded in this study and was widely distributed only in a few samples, such as Rongxian (RX-SC) and Nanchong (NC-SC).

The number of haplotypes, haplotype diversity (*Hd*), and nucleotide diversity (*Pi*) are used for assessing diversity in population-level COI sequencing surveys [42,43,44,45]. These metrics are useful for biodiversity assessment because they can be influenced by a variety of factors [46]. In the current study, the *Hd* and *Pi* values suggested that *P. tectus* may not be an ancient and stable population compared to *P. major*. The *Hd* and *Pi* among all *P. tectus* samples were lower than those of *P. major*. The *Hd* of *P. major* in Changfeng (CF-AH) was higher than in this study of other locations. In addition, the *Pi* of *P. major* in Changfeng (CF-AH) was up to 0.014. Among the *P. tectus* populations, the *Hd* and *Pi* of Changfeng (CF-AH) were the highest (*Hd* = 0.467, π = 0.001). Compared to *P. tectus*, the genetic variation of *P. major* was at a relatively high level, which was confirmed by allozyme loci [13,14].

Clonal diversity is often high in obligate asexual organisms and most research on asexual organisms has focused on the role of environmental heterogeneity in promoting clonal diversity [13,46,47]. The blue oat mite *P. major* is asexual and lacks a sexual relative. Populations consist of numerous clonal genotypes and clone frequencies change over time and space [13]. Previous studies have also provided direct evidence for negative frequency-dependent selection maintaining clonal variation in *P. major* [15]. In our research, haplotype 3 (MH3) was found in 17 *P. major* populations and haplotype 1 (TH1) was the most abundant in *P. tectus* populations. The different haplotypes represent different genetic backgrounds, and clone competition, differential resource utilization, and pesticide resistance likely cause the haplotypes’ diversity [48,49,50]. A larger haplotype variation was found in *P. major* than *P. tectus* populations, indicating that it has a stronger evolutionary potential for adaptation to future environmental changes and chemical pesticides.

We observed significant differentiation of *P. major* among regions, locations within regions, and within locations. The percentage of variation was only 2.05% between regions, but as much as 42.20% within locations. For *P. tectus*, the percentage of variation was up to 88.43% within locations. AMOVA analysis indicated a low level of genetic differentiation among regions of *P. major* (*F_CT_* = 0.02, *p* = 0.3323) and *P. tectus* (*F_CT_* = 0.02, *p* = 0.2063), which agrees with previous studies [14]. This may be due to the long-range movement of diapause eggs through winds or human assistance in summer [5,51].

## 5. Conclusions

In this study, the occurrence pattern of blue oat mites was clarified in three major winter wheat regions. The results indicated that the occurrence of blue oat mites was particularly serious in the Yellow and Huai River winter wheat zone of China, especially during the early spring season. Among the pest mites, *P. major* was found to be the most common in winter wheat, followed by *P. tectus*. Although *P. tectus* had not been previously reported in China, this study found that it occurred in all three wheat regions, and there was an evident co-occurrence between the *P. major* and *P. tectus*. Furthermore, the COI sequence analysis revealed that *P. major* boasts more genotypes and higher genetic diversity than *P. tectus*. It suggested that *P. major* may have stronger adaptability. Further studies should focus on exploring the tolerance of *P. major* and *P. tectus* to temperature, the fitness on different host plants, and the resistance to various chemicals. This way, we can develop a more reasonable scheme for regional precise control of these mites.

## Figures and Tables

**Table 1:** Sample collection information for *P. major* and *P. tectus* used in this study.

Region	Pop. ID	Population	Longitude (°E)	Latitude (°N)	Number of *P. major*	Number of *P. tectus*
SW (SC, CQ)	RX-SC	Sichuan, Rongxian	104°27′	29°25′	31	0
	MZ-SC	Sichuan, Mianzhu	104°14′	31°20′	30	0
	ST-SC	Sichuan, Santai	105°08′	31°10′	27	1
	NC-SC	Sichuan, Nanchong	106°05′	30°77′	2	29
	TN-CQ	Chongqing, Tongnan	105°84′	30°19′	32	0
YV (HB)	DY-HB	Hubei, Dangyang	111°55′	30°51′	10	16
	XZ-HB	Hubei, Xiangzhou	112°21′	32°07′	30	0
	ZY-HB	Hubei, Zaoyang	112°51′	32°12′	32	0
	ZX-HB	Hubei, Zhongxiang	112°36′	31°13′	4	0
YH (JS, AH, HN, SD, SX)	SN-JS	Jiangsu, Suining	117°95′	33°90′	31	0
	XZ-JS	Jiangsu, Xuzhou	117°14′	34°22′	28	2
	SY-JS	Jiangsu, Siyang	118°47′	33°46′	30	0
	DH-JS	Jiangsu, Donghai	118°29′	34°34′	3	1
	FN-AH	Anhui, Funan	115°58′	32°63′	31	0
	BZ-AH	Anhui, Bozhou	115°78′	33°85′	1	20
	CF-AH	Anhui, Changfeng	117°13′	32°26′	13	13
	XX-AH	Anhui, Xiaoxian	116°51′	34°12′	29	0
	DC-HN	Henan, Dancheng	115°07′	33°43′	22	0
	SZ-HN	Henan, Shanzhou	111°04′	34°43′	17	12
	TH-HN	Henan, Tanghe	112°44′	32°30′	17	13
	LH-HN	Henan, Luohe	113°54′	33°34′	31	0
	GT-SD	Shandong, Gaotang	116°25′	36°80′	18	0
	RC-SX	Shanxi, Ruicheng	110°68′	34°70′	0	1

The SW, YV, and YH stand for Southwestern China, Middle and Lower Yangtze Valleys, and Yellow and Huai Valleys, respectively.

**Table 2:** Variation analysis of COI gene sequences of *P. major* and *P. tectus*.

Species	Length of Sequences (bp)	C	V	PI	S	A + T (%)
*P. major*	657–658	636	22	19	3	70.30
*P. tectus*	657–658	654	4	4	None	69.90

C: Constant sites; V: variable sites; PI: Parsimony-informative sites; S: Singleton sites.

**Table 3:** Genetic diversity of *P. major* and *P. tectus* from different locations based on mitochondrial COI gene.

Species	Pop.ID	*H*	Haplotype (No. of Specimens)	*Hd*	*Pi*	*K*
*P. major*	MZ-SC	3	MH1 (14), MH2 (1), MH3 (15)	0.549	0.014	9.274
	ST-SC	4	MH1 (14), MH3 (11), MH4 (1), MH5 (1)	0.584	0.014	9.111
	NC-SC	1	MH1 (2)	0	0	0
	TN-CQ	3	MH1 (13), MH3 (17), MH6 (2)	0.567	0.014	9.085
	DY-HB	2	MH1 (4), MH3 (6)	0.513	0.014	9.231
	XZ-HB	1	MH3 (30)	0	0	0
	ZY-HB	1	MH3 (32)	0	0	0
	ZX-HB	1	MH1 (4)	0	0	0
	SN-JS	2	MH3 (30), MH7 (1)	0.065	0	0.065
	XZ-JS	1	MH3 (28)	0	0	0
	SY-JS	1	MH3 (30)	0	0	0
	DH-JS	1	MH3 (3)	0	0	0
	FN-AH	2	MH3 (30), MH8 (1)	0.065	0	0.065
	BZ-AH	1	MH1 (1)	0	0	0
	CF-AH	3	MH1 (4), MH3 (5), MH9 (4)	0.708	0.014	8.917
	XX-AH	2	MH3 (2), MH9 (27)	0.114	0.003	1.825
	DC-HN	2	MH1 (4), MH3 (18)	0.280	0.008	5.040
	SZ-HN	2	MH1 (15), MH9 (2)	0.221	0	0.441
	TH-HN	1	MH3 (17)	0	0	0
	LH-HN	3	MH1 (4), MH3 (9), MH9 (18)	0.581	0.011	7.277
	GT-SD	3	MH1 (14), MH3 (2), MH9 (2)	0.288	0.007	4.275
*P. tectus*	RX-SC	1	TH1 (31)	0	0	0
	ST-SC	1	TH1 (1)	0	0	0
	NC-SC	1	TH1 (29)	0	0	0
	DY-HB	2	TH1 (14), TH5 (2)	0.199	0.000	0.199
	XZ-JS	1	TH1 (2)	0	0	0
	DH-JS	1	TH1 (1)	0	0	0
	BZ-AH	2	TH1 (18), TH2 (2)	0.138	0.000	0.138
	CF-AH	4	TH1 (9), TH2 (1), TH3 (2), TH4 (1)	0.467	0.001	0.686
	SZ-HN	1	TH1 (12)	0	0	0
	TH-HN	1	TH1 (13)	0	0	0
	RC-SX	1	TH1 (1)	0	0	0

**Table 4:** Pairwise *F_ST_* (below diagonal) and gene flow *N_m_* (above diagonal) among 21 populations of *P. major* in winter wheat of China.

Locations	MZ- SC	ST- SC	NC- SC	TN- CQ	DY- HB	XZ- HB	ZY- HB	ZX- HB	SN- JS	XZ- JS	SY- JS	DH-JS	FN- AH	BZ- AH	CF- AH	XX- AH	DC- HN	SZ- HN	TH- HN	LH- HN	GT- SD
MZ-SC		inf	0.84	inf	inf	0.30	0.29	0.75	0.30	0.31	0.30	0.91	0.30	9.01	inf	0.41	1.62	0.31	0.40	2.80	0.64
ST-SC	−0.01		3.32	7.33	83.08	0.18	0.17	0.40	0.17	0.18	0.18	0.45	0.17	inf	inf	0.75	0.70	0.51	0.23	inf	1.36
NC-SC	0.23	0.07		0.55	0.56	0.00	0.00	0.00	0.00	0.00	0.00	0.00	0.00	inf	10.62	inf	inf	inf	inf	inf	inf
TN-CQ	−0.02	0.03	0.31		inf	0.41	0.40	1.15	0.41	0.43	0.41	1.50	0.41	1.36	7.56	0.31	3.13	0.25	0.55	1.50	0.46
DY-HB	−0.06	0.00	0.31	−0.07		0.21	0.20	1.11	0.21	0.22	0.21	1.67	0.21	2.00	inf	0.22	4.75	0.14	0.33	1.70	0.38
XZ-HB	0.45	0.59	1.00	0.38	0.55		inf	inf	inf	inf	inf	inf	inf	0.00	0.10	0.02	1.17	0.00	inf	0.11	0.02
ZY-HB	0.46	0.60	1.00	0.38	0.56	0.00		inf	249.8	inf	inf	inf	249.8	0.00	0.10	0.02	1.12	0.00	inf	0.11	0.02
ZX-HB	0.25	0.39	1.00	0.18	0.18	0.00	0.00		inf	inf	inf	inf	inf	0.00	0.33	0.04	inf	0.00	inf	0.23	0.06
SN-JS	0.46	0.59	1.00	0.38	0.55	0.00	0.00	−0.14		inf	inf	inf	inf	0.00	0.10	0.02	1.16	0.00	inf	0.11	0.02
XZ-JS	0.44	0.58	1.00	0.37	0.53	0.00	0.00	0.00	0.00		inf	inf	inf	0.00	0.11	0.02	1.24	0.00	inf	0.11	0.03
SY-JS	0.45	0.59	1.00	0.38	0.55	0.00	0.00	0.00	0.00	0.00		inf	inf	0.00	0.10	0.02	1.17	0.00	inf	0.11	0.02
DH-JS	0.22	0.36	1.00	0.14	0.13	0.00	0.00	0.00	−0.02	0.00	0.00		inf	0.00	0.38	0.04	inf	0.00	inf	0.23	0.06
FN-AH	0.46	0.59	1.00	0.38	0.55	0.00	0.00	−0.14	0.00	0.00	0.00	−0.02		0.00	0.10	0.02	1.16	0.00	inf	0.11	0.02
BZ-AH	0.03	−0.23	0.00	0.16	0.11	1.00	1.00	1.00	1.00	1.00	1.00	1.00	1.00		inf	inf	0.15	inf	0.00	inf	inf
CF-AH	−0.01	−0.06	0.02	0.03	0.00	0.71	0.72	0.43	0.71	0.70	0.71	0.40	0.71	−0.33		0.72	0.59	0.41	0.15	inf	1.59
XX-AH	0.38	0.25	−0.30	0.45	0.54	0.93	0.93	0.88	0.93	0.93	0.93	0.87	0.93	−0.93	0.26		0.09	27.53	0.02	1.80	inf
DC-HN	0.13	0.26	0.66	0.07	0.05	0.17	0.18	−0.03	0.18	0.17	0.18	−0.08	0.18	0.62	0.30	0.73		0.07	1.85	0.36	0.14
SZ-HN	0.44	0.33	0.00	0.50	0.64	1.00	1.00	1.00	1.00	1.00	1.00	1.00	1.00	0.00	0.38	0.01	0.79		0.00	0.97	4.8
TH-HN	0.38	0.52	1.00	0.31	0.43	0.00	0.00	0.00	−0.02	0.00	0.00	0.00	−0.02	1.00	0.62	0.91	0.12	1.00		0.14	0.03
LH-HN	0.08	0.00	−0.07	0.14	0.13	0.70	0.70	0.54	0.70	0.69	0.70	0.52	0.70	−0.47	−0.04	0.12	0.41	0.21	0.64		5.18
GT-SD	0.28	0.16	−0.27	0.35	0.40	0.91	0.91	0.81	0.91	0.91	0.91	0.80	0.91	−0.88	0.14	−0.04	0.65	0.05	0.88	0.05	

**Table 5:** Pairwise *F_ST_* (below diagonal) and gene flow *N_m_* (above diagonal) among 10 populations of *P. tectus* in winter wheat of China.

Locations	RX-SC	ST-SC	NC-SC	XZ-JS	BZ-AH	CF-AH	SZ-HN	TH-HN	RC-SX	DY-HB
RX-SC		inf	inf	inf	2.63	0.58	inf	inf	inf	1.63
ST-SC	0.00		inf	inf	inf	inf	inf	inf	inf	inf
NC-SC	0.00	0.00		inf	2.83	0.62	inf	inf	inf	1.74
XZ-JS	0.00	0.00	0.00		inf	inf	inf	inf	inf	inf
DH-JS	0.00	0.00	0.00	0.00		inf	inf	inf	inf	inf
BZ-AH	0.09	−0.89	0.08	−0.28		2.35	15.00	11.04	inf	3.76
CF-AH	0.30	−0.67	0.29	−0.16	0.10		1.35	1.25	inf	1.3
SZ-HN	0.00	0.00	0.00	0.00	0.02	0.16		inf	inf	5.79
TH-HN	0.00	0.00	0.00	0.00	0.02	0.17	0.00		inf	4.92
RC-SX	0.00	0.00	0.00	0.00	0.00	−0.89	−0.67	0.00		inf
DY-HB	0.13	−0.87	0.13	−0.27	0.06	0.16	0.04	0.05	-0.87	

**Table 6:** Pairwise *F_ST_* (below diagonal) and gene flow *N_m_* (above diagonal) among different geographic regions of *P. major* based on mitochondrial.

Locations	Southwestern China (SW)	Middle and Lower Yangtze Valleys (YV)	Yellow and Huai Valleys (YH)
Southwestern China (SW)		0.43819	1.08303
Middle and Lower Yangtze Valleys (YV)	0.36327		3.81602
Yellow and Huai Valleys (YH)	0.18754	0.06149	

**Table 7:** Pairwise *F_ST_* (below diagonal) and gene flow *N_m_* (above diagonal) among different geographic regions of *P. tectus* based on mitochondrial.

Locations	Southwestern China (SW)	Middle and Lower Yangtze Valleys (YV)	Yellow and Huai Valleys (YH)
Southwestern China (SW)		4.23242	0.84639
Middle and Lower Yangtze Valleys (YV)	0.05577		4.07018
Yellow and Huai Valleys (YH)	0.22802	0.05787	

**Table 8:** Analysis of molecular variance (AMOVA) of COI gene sequences in different geographic regions of *P. major* and *P. tectus*.

Species	Source of Variation	df	Sum of Squares	% of Variation	Fixation Indices	*p*-Value
*P. major*	Among regions	2	152.02	2.05	F_CT_ = 0.02	*p* = 0.332
	Among locations within regions	18	859.49	55.74	F_SC_ = 0.57	*p* < 0.001
	Within locations	417	733.64	42.20	F_ST_ = 0.58	*p* < 0.001
*P. tectus*	Among regions	2	0.53	2.35	F_CT_ = 0.02	*p* = 0.206
	Among locations within regions	8	1.04	9.21	F_SC_ = 0.09	*p* = 0.239
	Within locations	128	8.17	88.43	F_ST_ = 0.12	*p* = 0.070

## Data Availability

The data generated during the study have already been reported in the manuscript.
